# Assessing Awareness, Protective Eyewear Utilization, and Associated Factors Among Construction Workers in Nagpur, Maharashtra

**DOI:** 10.7759/cureus.111254

**Published:** 2026-06-21

**Authors:** Rajesh Pattebahadur, Kanishk Singh, Sagarika Snehi, Pooja Panjwani, Vandana Iyer

**Affiliations:** 1 Ophthalmology, All India Institute of Medical Sciences, Nagpur, IND; 2 Ophthalmology, Dr Agarwals Eye Hospital, Mumbai, IND

**Keywords:** community education, community ophthalmology, construction industry, epidemiology of ocular trauma, eye injury prevention, survey research

## Abstract

Background and aim

Workers on construction sites and in multiple industries experience many ocular injuries, which can affect their ability to continue working. Such injuries can be easily prevented with appropriate and effective eye protection. This study aimed to evaluate the awareness of occupational ocular injury risks and their potential implications, such as visual impairment and blindness, among construction workers (knowledge domain); assess worker attitudes regarding occupational ocular safety and the utilization of protective eyewear (attitude domain); assess the prevalence of protective eyewear usage and identify obstacles to its adoption among construction workers (practice domain); and determine the independent variables of protective eyewear utilization, including educational level, exposure to occupational hazards, length of work, and history of previous ocular injury, using multivariable logistic regression analysis.

Methods

A cross-sectional analytical study was conducted among 133 participants who presented to the All India Institute of Medical Sciences (AIIMS), Nagpur Ophthalmology OPD, and were engaged in various work at construction sites in and around Nagpur, Maharashtra. Data were collected over a period of 12 months from January 2022 to December 2022. Participants were given an expert-reviewed, content-validated, bilingual (English and Hindi) questionnaire, and responses were recorded by the investigator on a predesigned data collection form. Literate participants independently completed the structured bilingual questionnaire, while illiterate participants had each question read aloud in Hindi by the investigator, and their responses were recorded on a predesigned proforma. All statistical tests were performed with a 95% confidence interval, and p < 0.05 was considered statistically significant.

Results

A total of 133 participants were enrolled in the study. Nearly all respondents (132; 99.2%) knew that their job could cause eye injury. Upon analyzing education level, seven (15.2%) primary school-educated workers wore protection, compared with 17 (56.7%) post-high school-educated workers and three (50%) diploma holders. Educational status was significantly associated with the use of protective eye gear (p = 0.003). Educational status (adjusted odds ratios {AOR} = 2.865, 95% CI: 1.725-4.739, p < 0.001), type of work (AOR = 0.707, 95% CI: 0.549-0.911, p = 0.007; interpreted with caution given nominal scaling of work categories), and prior injury history (AOR = 3.886, 95% CI: 1.472-10.257, p = 0.006) were significant independent predictors of protective eyewear use. Duration of work was not a significant independent predictor of protective eyewear use (AOR = 0.864, 95% CI: 0.458-1.629, p = 0.650).

Conclusion

This study concluded that although workers' awareness of the use of protective eye gear was high, adherence and use were very low. Educational status, type of work, and prior history of ocular injury were key determinants of protective eyewear use.

## Introduction

The construction work all over the world includes land preparation, construction, welding, plumbing, cementing, tile work, electrical work, etc. Workers engaged in these activities are exposed to various occupational hazards. According to a 2017 report by the Centre for Policy Research (CPR), India, workers from the construction industry constitute 7.5% of the total world labor force [1]. In India, the prevalence of occupational hazards in the construction sector is attributed to several factors, including heavy dependence on manual labor, inadequate regulatory enforcement, insufficient worker training, low awareness of workplace hazards, and poor compliance with occupational safety measures among workers [1].

Occupational eye injuries are a preventable cause of ocular morbidity globally, with construction workers being susceptible to exposure to mechanical, chemical, and environmental hazards [1,2]. As per Samanta and Gochhayat, occupational hazards in India range from 924,700 to 1,902,300 cases per year, with approximately 121,000 deaths annually [2]. Among them, on construction sites, approximately 47.4% of construction workers faced occupational hazards [2]. This portrays the poor working conditions of construction workers and their risk of exposure to occupational hazards. In India, most workers are from remote villages with a poor educational background and poor awareness about occupational hazards. Despite the significant contribution of the construction sector to India's economy, there remains a notable lack of published data on occupational health hazards, worker awareness, and preventive strategies in this population. The present study involved construction workers in Nagpur, Maharashtra. This study aimed to evaluate the awareness of occupational ocular injury risks and their potential implications, such as visual impairment and blindness, among construction workers (knowledge domain); assess worker attitudes regarding occupational ocular safety and the utilization of protective eyewear (attitude domain); assess the prevalence of protective eyewear usage and identify obstacles to its adoption among construction workers (practice domain); and determine the independent variables of protective eyewear utilization, including educational level, exposure to occupational hazards, length of work, and history of previous ocular injury, using multivariable logistic regression analysis.

## Materials and methods

Study design

This was a cross-sectional analytical study conducted at the Ophthalmology OPD of the All India Institute of Medical Sciences (AIIMS), Nagpur. For literate participants, written informed consent was obtained directly. For illiterate participants, the consent form was read aloud in the local vernacular in the presence of a literate, impartial witness, who co-signed it on their behalf.

Inclusion and exclusion criteria

The study included workers aged 18 years or older engaged in various construction-related occupations, including hammer-chisel work, welding, painting, drilling, stone crushing, and other allied construction activities, who attended the ophthalmology OPD at AIIMS Nagpur for routine examinations or treatment of ocular injuries. Participants under 18 years of age and those unwilling to provide consent were excluded from the study.

A structured, expert-reviewed, content-validated, bilingual (English and Hindi) questionnaire was used as the study tool. Hindi was used as it was the most commonly understood language among the study participants. A questionnaire was developed after a review of published literature on occupational eye hazards and protective eyewear practices among welders [3]. The questionnaire consisted of the following three domains: (1) demographic profile-age, educational qualification, and duration of employment, (2) awareness of occupational eye hazards-knowledge of injury risk, potential for blindness, preventive measures, and appropriate first-aid responses, and (3) eye protection practices-availability, frequency of use, and barriers to the use of protective eyewear. An expert review by faculty members of the Department of Ophthalmology, AIIMS Nagpur, was conducted prior to finalization to ensure content validity. However, formal psychometric validation, including pilot testing, internal consistency assessment, and test-retest reliability, was not performed; this shortcoming is acknowledged as a limitation of this study. Each questionnaire took approximately 10-15 min to complete. Data collection was carried out over 12 months, from January 2022 to December 2022. The complete bilingual questionnaire is provided in appendix.

Sample Size Calculation

According to literature from Budhathoki et al., 90% of workers were aware of ocular injuries and the importance of using protective devices [3]. Thus, with a prevalence (p) of 90%, an alpha error of 0.05, an estimated proportion (p) of 0.90, and an estimated error of 0.05, a sample size of 139 was calculated.

Data collection and statistical analysis

Data were entered in Microsoft Excel 2013 (Redmond, WA: Microsoft Corp.) and analyzed using IBM SPSS Statistics version 21 (Armonk, NY: IBM Corp.). Descriptive statistics were employed to summarize the study variables as frequencies and percentages. Demographic, awareness, and workplace practice variables are presented descriptively, as they represent sample characteristics rather than hypothesis-driven comparisons. Inferential statistics were used for testing associations between independent variables and the primary outcome of protective eyewear use. The chi-square test of independence was employed to examine relationships between categorical variables when the test's assumptions were satisfied. Fisher's exact test was used when expected cell counts were fewer than five, especially in weakly filled contingency tables. For questions that allowed multiple responses, participants could select more than one option. For such responses, no chi-square or other inferential statistics were applied, as responses to such questions are not mutually exclusive; they violate the independence assumption required for inferential testing of multiple-response items, and data are presented as frequencies and percentages only. The relationship between tenure of work and use of protective eyewear was determined using Spearman's rank correlation coefficient. Although protective eyewear use was recorded as a binary variable, it was treated as the lower bound of an ordinal scale for the purpose of Spearman's rank correlation. This analysis was performed in addition to the chi-square test of independence and multivariable logistic regression, both of which corroborate the non-significant association between duration of work and eyewear use. Independent determinants of protective eyewear use were determined using binary logistic regression analysis. Protective eyewear use (yes/no) was the dependent variable. The outcome variable was coded as protective eyewear use (1 = yes, 0 = no); therefore, adjusted odds ratios (AORs) > 1 indicate higher odds of protective eyewear use, consistent with conventional logistic regression reporting. Independent variables were educational level, kind of occupation, duration of employment, and previous history of eye injury. Educational level was entered into the model as an ordinal variable, with higher values indicating a higher level of education. The type of work was also entered as an ordinal variable in the logistic regression model, with welding assigned the highest code based on an assumed hierarchy of occupational hazard exposure. It is acknowledged that these work categories are inherently nominal in nature and do not represent a validated ordinal scale. The use of ordinal coding for this variable is a methodological limitation; the resulting AOR reflects an overall linear trend across the assumed ordering rather than true category-specific effects. Unadjusted odds ratios (UOR) and adjusted odds ratios (AOR) with 95% confidence intervals (CI) were estimated. All analyses were performed at a 95% confidence level, and p < 0.05 was considered statistically significant.

## Results

Demographic distribution of participants

A total of 139 eligible participants were approached for enrollment. Six participants declined consent and were excluded as per predefined exclusion criteria. Therefore, data from 133 participants were included in the final analysis. The non-response rate was low (4.3%) and unlikely to significantly affect the study findings; males comprised 113 (85%), whereas females made up only 20 (15%), indicating a male-dominated workforce. The participants were almost equally divided into urban (65; 48.9%) and rural (68; 51.1%) regions, showing no significant difference. Regarding marital status, 68 (51.1%) were not married, and 65 (48.9%) were married, with no strong association. In relation to educational qualification, the majority of the participants had primary school education (46; 34.6%), or high school education (45; 33.8%), followed by post-high school education (class 11 and 12/higher secondary certificate level) (30; 22.6%), some being illiterate (six; 4.5%), or diploma holders (six; 4.5%) (Table 1).

**Table 1:** Distribution of the demographics of the participants (n = 133). Data presented as frequencies and percentages. No inferential statistics were applied, as these represent descriptive sample characteristics. High school certificate - equivalent to completion of class 10. Post-high school education - equivalent to completion of classes 11 and 12.

Variables	Category	Frequency	Percentage
Gender	Male	113	85
	Female	20	15
Urban/rural	Urban	65	48.9
	Rural	68	51.1
Marital status	Bachelors/unmarried	68	51.1
	Married	65	48.9
Education	Illiterate	6	4.5
	Primary school certificate	46	34.6
	High school certificate	45	33.8
	Post high school	30	22.6
	Diploma	6	4.5
	Graduate	0	0

Awareness of ocular injury and work profile

Nearly all respondents (132; 99.2%) knew that their job could cause eye injury; this near-unanimous distribution precluded the application of inferential statistics, and data are presented descriptively only. Likewise, 96 (72.2%) participants appropriately identified that such an injury could cause blindness, indicating good awareness of ocular hazards. For preventive actions, 70 (52.6%) chose more than one option, including eye protection and precautions, and 61 (45.9%) individually mentioned eye protection devices. Regarding awareness of different protective eye devices, 122 (91.7%) selected more than one correct option, including goggles, face shields, and professional gear, indicating good knowledge of protective gear. In the event of ocular injury, 66 (49%) participants preferred washing the eyes, whereas the other 67 (51%) preferred more than one first-aid intervention. Due to the multiple-response format of this item, inferential statistics were not applied, and data are presented descriptively. Regarding healthcare-seeking behavior, 86 (64.7%) preferred visiting a healthcare provider available and close to them, including an eye specialist, whereas 47 (35.3%) preferred only an ophthalmologist. Among diverse work types, the majority were engaged in hammer-chisel work (31; 23.3%), followed by painting (25; 18.8%), welding (24; 18%), and others (26; 19.5%), with considerable differences. With respect to duration of employment, 56 (42.1%) were employed for less than one year, 56 (42.1%) were employed for one to five years, and 21 (15.8%) were employed for more than five years, indicative of a relatively new and transitory workforce (Table 2).

**Table 2:** Knowledge and awareness regarding occupational ocular injuries, blindness, and eye protection measures among study participants (n = 133). #Denotes multiple-response items; percentages reflect the distribution of response categories and do not sum to 100%. Data presented as frequencies and percentages. No inferential statistics were applied, as these items represent descriptive characterization of awareness and work profile domains. h/o: history of; FB: foreign body; IOFB: intraocular foreign body; EOFB: extraocular foreign body

Variables	Response	Frequency	Percentage
Can your work lead to eye injury	Yes	132	99.2
	No	1	0.8
	Not sure	0	0
Can such injury lead to blindness	Yes	96	72.2
	No	0	0
	Not sure	37	27.8
Ocular injury at your workplace can be prevented by which of following#	Precautions	2	1.5
	Eye protection	61	45.9
	Precautions, eye protection, keep distance, with experience	70	52.6
What are various eye protection devices#	Normal goggle/glass/spectacle	1	0.8
	Specialized eye protection gear/glass	1	0.8
	Face shield	9	6.8
	Specialized eye protection gear/glass, face shield	122	91.7
What should be done in case of ocular injury#	Eye wash	66	49
	Eye wash, rubbing of eyes, and over-the-counter medications	67	51
Which doctor, according to you, should be consulted after injury#	Eye specialist	47	35.3
	Not needed; general physician, ayurveda/homeopathic practitioners	86	64.7
Type of work	Hammer-chisel	31	23.3
	Welding	24	18
	Painting	25	18.8
	Drilling	12	9
	Stone crushing	15	11.3
	Others	26	19.5
Duration of work	Less than 1 year	56	42.1
	1-5 years	56	42.1
	More than 5 years	21	15.8
Past h/o FB injury	Yes	41	30.8
	No	92	69.2
If yes, how many times	1/1	26	63.4
	2/2	14	34.1
	3/3	1	2.4
	Total	41	100
H/o self-medication for the past injury	Yes	5	12.2
	No	36	87.8
	Total	41	100
Type of FB	IOFB	1	2.4
	EOFB	40	97.6
	Total	41	100
Time duration between injury and doctor's visit	<24-h	32	78
	24-72 h	8	19.5
	>72 h	1	2.4
	Total	41	100
Treating doctor	Ophthalmologist	31	75.6
	Non-ophthalmologist	10	24.4
	Total	41	100

History of injury to other body parts

A total of 18 (13.5%) workers had injuries to body parts other than the eyes, with hands being the most frequently injured site (12; 9%), followed by feet (three; 2.3%), fingers (two; 1.5%), and shoulders (one; 0.8%). The majority of workers (115; 86.5%) did not receive treatment for their injuries, while 13 (9.8%) received hospital treatment, and five (3.8%) used self-medication (Table 3).

**Table 3:** Profile of injury to other body parts among participants (n = 133). Data are presented as frequencies and percentages. These variables represent descriptive injury profiles of the sample and are not subject to inferential testing. The affected body part was reported only among injured participants (n = 18).

Variables	Response	Frequency	Percentage
History of injury to other body part	Yes	18	13.5
	No	115	86.5
If yes, specify body part affected	Hand	12	9
	Fingers	2	1.5
	Shoulder	1	0.8
	Foot	3	2.3
Which treatment taken for the injury	Self-medication	5	3.8
	Hospital treatment	13	9.8

Workplace safety practices

A high number of participants (112; 84.2%) reported being offered eye-protective equipment at work, but only 42 (31.6%) used it, indicating a substantial gap between provision and utilization. The distribution of reasons for non-use of protective eyewear varied across categories, with some barriers more prevalent than others. The most common reasons reported for non-use of protective eyewear were poor visibility (32; 24.1%) and difficulty working (26; 19.5%), followed by non-availability (17; 12.8%) and poor fit (10; 7.5%). Due to multiple cells with expected frequencies below five, inferential statistics were not applied to this variable, and data are presented descriptively (Table 4). When divided by gender, 38 (33.6%) men and four (20%) women wore protective eyewear, with no significant difference (p = 0.301) (Table 5).

**Table 4:** Use of protective eye gear. *Multiple-response item; percentages reflect distribution of response categories and do not sum to 100%. Data presented as frequencies and percentages. Provision and use of eyewear are presented descriptively.

Variables	Responses	Frequency	Percentage
Provided with eye protective devices at the workplace?	Yes	112	84.2
	No	21	15.8
Use of eyewear?	Yes	42	31.6
	No	91	68.4
Reason for not using*	No perceived need	3	2.3
	Poor fitting	10	7.5
	Low-risk task	0	0
	Short duration of task	1	0.8
	No availability	17	12.8
	Difficulty in working	26	19.5
	Poor visibility	32	24.1
	Forgetfulness	0	0
	Hurry	0	0
	Cost	2	1.5
	Others	0	0

**Table 5:** Comparison of the usage of protective eyewear between gender (Fisher's exact test). P < 0.05 was considered statistically significant.

Gender		Usage of protective eyewear		Total	p-Value
		Yes	No		
Male	n	38	75	113	0.301
	%	33.6	66.4	100	
Female	n	4	16	20	
	%	20	80	100	
Total	n	42	91	133	
	%	31.6	68.4	100	

Comparison of protective eyewear by demographics

In terms of level of education, seven (15.2%) primary school-educated workers wore protection, compared with 17 (56.7%) post-high school-educated workers and three (50%) diploma holders. Work experience did not have a significant impact on the use of eyewear (p = 0.816). Similarly, knowledge of one's susceptibility to eye injury did not significantly affect eyewear use (p = 0.684).

The overall association between educational level and protective eyewear use was statistically significant (p = 0.003). However, category-specific odds ratios were imprecisely estimated with wide confidence intervals, reflecting small subgroup sample sizes, as detailed in the predictors section below (Table 6).

**Table 6:** Comparison of the usage of protective eyewear and education (Fisher's exact test). The reference category is illiterate.

Education		Usage of protective eyewear		Total	Unadjusted OR (95% CI)	p-Value
		Yes	No			
Illiterate	n	1	5	6	1 (reference category)	0.003
	%	16.7	83.3	100		
Primary school certificate	n	7	39	46	0.90 (0.09-8.89)	
	%	15.2	84.8	100		
High school certificate	n	14	31	45	2.26 (0.24-21.17)	
	%	31.1	68.9	100		
Post high school	n	17	13	30	6.54 (0.68-62.99)	
	%	56.7	43.3	100		
Diploma	n	3	3	6	5 (0.34-72.77)	
	%	50	50	100		
Total	n	42	91	133	-	
	%	31.6	68.4	100		

Predictors of protective eyewear usage

Univariate analysis was performed to calculate unadjusted odds ratios (UORs) for the association between the use of protective eyewear and the chosen explanatory variables. Binary logistic regression was performed to identify independent predictors of protective eyewear use, adjusting for education, occupation type, employment duration, and history of eye injury. Higher educational status was a significant positive predictor of eyewear use (AOR = 2.865, 95% CI: 1.725-4.739, p < 0.001), indicating that workers with higher education were approximately 2.9 times more likely to use protective eyewear. The correlation between educational level and use of protective eyewear in general was statistically significant (p = 0.003). However, the category-specific risk ratios were imprecisely computed, with broad confidence intervals spanning unity, reflecting likely small subgroup sample sizes. Even though the association between educational level and protective eyewear use was statistically significant (p = 0.003), all confidence intervals crossed 1.0. None of the category-specific unadjusted odds ratios reached individual statistical significance. A trend towards higher odds of protective eyewear use was observed with increasing educational level. The workers with post-high-school education showed the highest unadjusted odds compared with illiterate workers (OR: 6.54, 95% CI: 0.68-62.99), followed by diploma holders (OR: 5.00, 95% CI: 0.34-72.77). These estimates are imprecise due to small subgroup sample sizes and should be interpreted with caution. Type of work was a significant predictor of protective eyewear use (AOR = 0.707, 95% CI: 0.549-0.911, p = 0.007). As a type of work was entered as an ordinal variable, with welding assigned the highest code, an AOR of 0.707 indicates that workers in higher-coded occupational categories had lower odds of eyewear use. This estimate reflects an overall ordinal trend and should be interpreted with caution. Workers with a past history of ocular injury had approximately 3.9 times higher odds of using protective eyewear compared to those without such a history (AOR = 3.886, 95% CI: 1.472-10.257, p = 0.006), indicating that prior injury experience promotes protective behavior. Duration of work was not a significant independent predictor of eyewear use (AOR = 0.864, 95% CI: 0.458-1.629, p = 0.650) (Table 7).

**Table 7:** Predictors of protective eyewear use among construction workers - binary logistic regression analysis (n = 133). Outcome variable coded as eyewear use = 1 (yes), non-use = 0 (no); therefore, AOR > 1 indicates increased likelihood of protective eyewear use, and AOR < 1 indicates decreased likelihood. Education was entered as an ordinal variable with higher values indicating a higher educational level. The type of work was entered as an ordinal variable with higher values reflecting greater occupational hazard exposure. All analyses were performed at 95% confidence level. P < 0.05 was considered statistically significant. B: regression coefficient; SE: standard error; Wald: Wald statistic; df: degree of freedom; AOR: adjusted odds ratio

Variables	B	SE	Wald	df	p-Value	AOR	95% CI (lower)	95% CI (upper)
Education	1.052	0.257	16.679	1	<0.001	2.865	1.725	4.739
Type of work	-0.347	0.129	7.220	1	0.007	0.707	0.549	0.911
Duration of work	-0.147	0.324	0.206	1	0.650	0.864	0.458	1.629
Past history of injury	1.357	0.495	7.514	1	0.006	3.886	1.472	10.257
Constant	-0.375	1.242	0.091	1	0.762	0.687	-	-

Correlation between eyewear usage and duration of work

**Figure 1:**
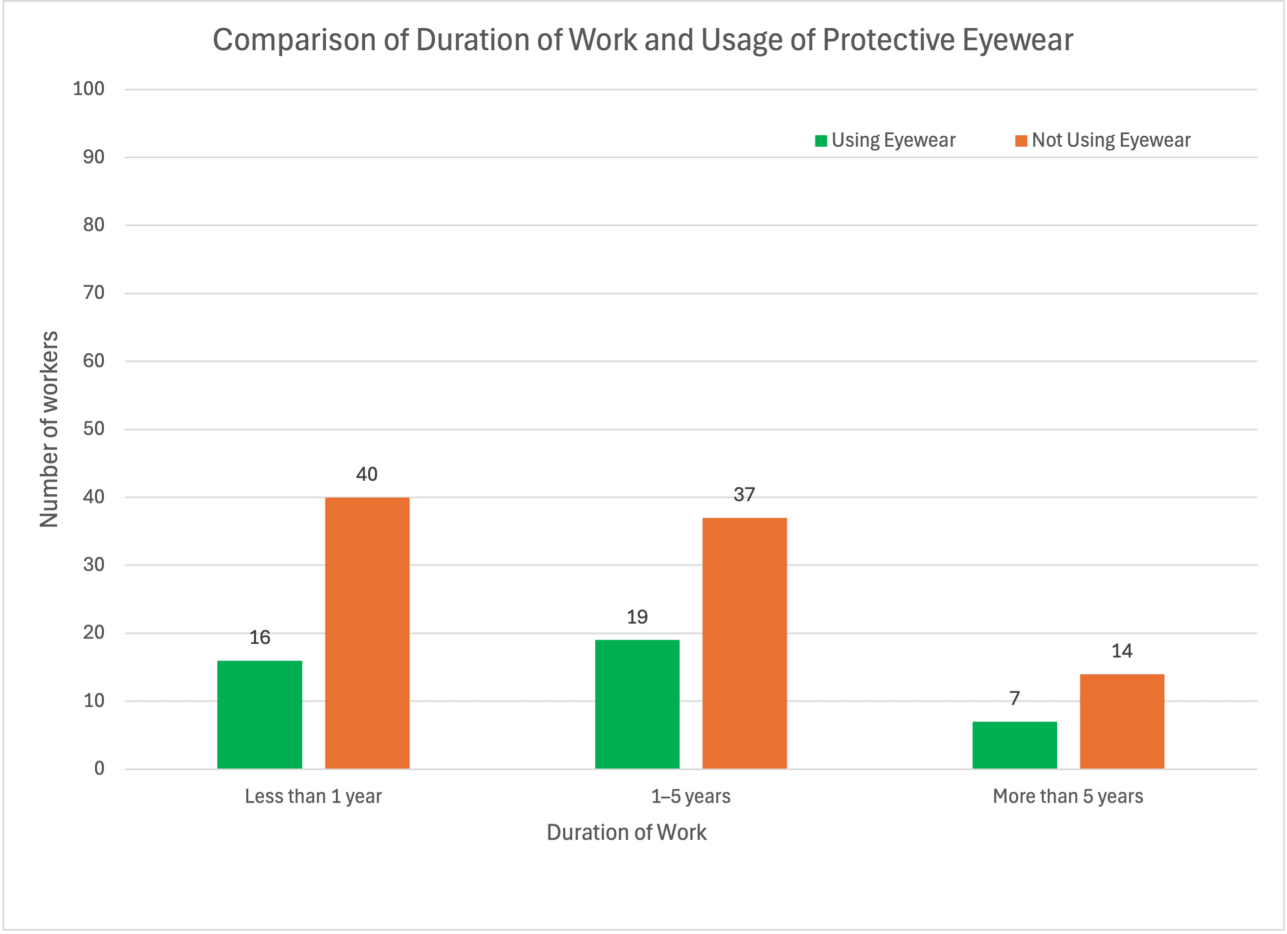
Clustered bar chart showing the number of construction workers using and not using protective eyewear across duration of work categories (n = 133).

The relation between work duration and protective eyewear usage was weak and statistically insignificant (Table 8). Spearman's rank correlation coefficient was used to test the link between duration of work and usage of protective eyewear. The analysis showed a very weak, negative, and statistically non-significant correlation between the two variables (rₛ = -0.050, p = 0.570, n = 133). This suggests that the duration of work was not significantly associated with the use of protective eyewear among construction workers. Longer employment was not associated with an increased or decreased likelihood of protective eyewear use compared to shorter work experience, as illustrated in Figure 1.

**Table 8:** Comparison of the usage of protective eyewear between duration of work (chi-square test of independence). P < 0.05 was considered statistically significant.

Duration of work		Usage of protective eyewear		Total	p-Value
		Yes	No		
Less than 1 year	n	16	40	56	0.816
	%	28.6	71.4	100	
1-5 years	n	19	37	56	
	%	33.9	66.1	100	
More than 5 years	n	7	14	21	
	%	33.3	66.7	100	
Total	n	42	91	133	
	%	31.6	68.4	100	

## Discussion

The present study revealed a large gap between knowledge and behavior; although almost all workers knew about the risk of occupational eye injury (99.2%), only 31.6% reported using eye protection. Higher educational status, history of ocular injury, and type of work were important independent predictors of eyewear use, whereas duration of work was not. These results suggest that knowledge alone may not be sufficient to promote the adoption of protective eye safety procedures among construction workers and highlight the need for interventions to increase compliance with protective eyewear use.

The majority of participants in our study were male; similar results were reported in other studies, in which most participants were male [1,2,4-7]. However, in the studies conducted by Chauhan et al. [8], Prabhu et al. [9], and Sehsah et al., all participants were male, reflecting male dominance in labor-intensive work [10]. In our study, the involvement of rural and urban workers was equal, with a distribution similar to that reported by Prabhu et al. [9]. The majority of participants had fewer than five years of experience, indicating a relatively young and less experienced population involved in the construction business. A study in the UAE by AlMahmoud et al. on industrial workers in small-sized firms also reported that participants had limited experience, with an average tenure of four years [11]. Furthermore, a Malaysian study on construction workers by Ulang et al. reported an average duration of labor of three years [12], which was also supported by previous studies [5]. This type of group tends to have lower levels of education and no formal training for the tasks they perform, which would put them at greater risk of reduced adherence to protective gear and a higher incidence of overall occupational injuries, including ocular injuries [3,13].

The result of our study showed that a large number of participants were aware of the risk of ocular injury in their jobs. About 99% of participants in our study showed awareness of the risk of injury. This was similar to the study by Budhathoki et al., which tested awareness of occupational hazards among welders and found 90% awareness among participants [3]. Similarly, in Zimbabwe, Kwarteng et al. found that 99% of welders were aware of the eye hazards associated with their work [14]. In Nigeria, Ajayi et al. reported similar results, with 90% of welders aware of occupational hazards and using protective eye devices [15], similar to an Indian study by Prabhu et al. and an UAE study by AlMahmoud et al., wherein 95% of participants were aware of the risky nature of their job and the need for the use of preventive eye gear [9,11]. This was in contrast to a study by Ulang et al., where only 84% of participants were aware of protective gear and its importance [12]. This difference was due to prior on-site exposure and training for workers. All these studies imply that all workers, regardless of their profession, have a higher level of risk awareness and use of preventive gear against ocular injuries. Although the awareness level was high, the utilization of protective equipment was low; in our study, only 31.6% used it during the actual work. In our study, workers engaged in high-risk work, such as welding, had a higher probability of using protective gear. This was similar to a study by Budhathoki et al. in Nepal, where only 47.7% of participants and 15.5% in a study by Kwarteng et al. in Zimbabwe were not using protective gear while at work, and in the Nigerian study by Ajayi et al., 38% of participants were not using protective gear [3,14,15]. The differences between these could be due to our study including all types of workers, whereas other studies focused on welders. As welding is a high-risk job, a higher percentage of use of protective gear among study participants was seen. A similar trend of low use of eye protective gear was observed in other studies [4,5,9,11]. The main reason for non-utilization of protective gear in our study was poor visibility (24.1%); similarly, Lette et al. and AlMahmoud et al. reported the same reason in 75% and 70% of the participants, respectively [6,11]. In a Nigerian study by Kwarteng et al. in Zimbabwe and a study by Ajayi et al., 22.4% of participants did not use them because of discomfort during use [14,15]. In our study, 2.3%; in Prabhu et al.'s study, 20%; and in AlMahmoud et al.'s study, 10% of participants reported not feeling the need to use protective gear [9,11]. However, some studies found non-availability of protective gear to be the main reason for non-utilization [4,5,7]. This difference might be due to loose safety protocols in the respective countries.

In our study, one-third of participants had a history of ocular injuries. Additionally, 13.5% had a history of injury to other body parts, such as hands, feet, shoulders, and fingers. The literature has reported a 30-60% rate of injury to various body parts among different types of workers, including construction workers, small industrial workers, and welders [4-7,10].

For preventive actions, more than half of the participants in our study chose more than one option, including eye protection and precautions, and 45.9% of participants mentioned eye protection devices alone. While over half of the respondents selected multiple preventive options, they chose the less reliable or incorrect measures within these responses. These findings are suggestive that awareness of methods to prevent ocular injury is not uniformly evidence-based. This indicates a partial understanding among participants rather than scientific knowledge of how to prevent occupational hazards. A similar conclusion was reached in a Malaysian study, in which participants who were construction site workers did not have comprehensive knowledge, but they had a good understanding of the chances of injury, its preventive measures, and primary treatment in case of injury [12]. Multiple studies emphasized the need for formal training for workers to raise awareness of occupational hazards and to provide scientifically validated ways to prevent them [6,7,10].

Half of the participants from our study chose eyewashing in the event of ocular injury, whereas the other half selected multiple options, such as rubbing the eyes and using over-the-counter medication. These selection patterns explain that the participants were aware of the need for immediate intervention in the event of injury. Although such improper first-aid procedures may not be entirely effective, eyewashing is a suitable first response in many situations.

Among the participants who had a history of injury while doing work, they sought medical care within the first 24 h, and most of them got treatment from an ophthalmologist. The participants showed a positive attitude towards seeking proper medical care. This was due to the proper treatment provided by the authority under which the workers were employed.

Higher education, history of prior ocular injury, and high-risk work profiles, such as welding, were significant independent predictors of protective eyewear use. Workers with higher education were nearly three times more likely to use protective eyewear, consistent with findings of the study by Budhathoki et al. [3], Kwarteng et al. [14], and Ajayi et al., who similarly reported that educational level was positively associated with protective device use [15]. Workers with a history of ocular injury were approximately four times more likely to use eyewear, suggesting that lived experience of injury heightens risk perception and promotes protective behavior. It is acknowledged that work type categories are nominally scaled, and their treatment as an ordinal variable in the regression model is a methodological limitation. The reported AOR should therefore be interpreted as reflecting an assumed ordinal trend rather than a validated hierarchical relationship between work categories. Duration of employment was not a significant predictor, consistent with the non-significant Spearman's correlation (rₛ = -0.050, p = 0.570). A study by Sehsah et al. found that older participants aged more than 35 years and literate had more chances of complying with protective gear use [10]. All but one of the participants believed that there was a danger of eye harm in their employment. He was an electrician. He said the work carried little risk of eye damage but a greater chance of injury from electrical shock.

The findings of this study have substantial implications for clinical practice and public health. Most of the workers (99.2%) are aware of the necessity of protective eyewear; however, most of them (68.4%) are not utilizing it. This highlights the need for immediate, comprehensive safety actions on construction sites. Workers who had previously been injured in the eye were more likely to wear protective eyewear (AOR = 3.886), suggesting that safety programs should be implemented before injuries occur. Ophthalmologists and occupational health practitioners should advocate the necessity for compulsory eye safety training, especially for less-educated, lower-risk workers with lower compliance rates. Another concern with protective eyewear is poor visibility and discomfort when working. It’s the responsibility of employers and the government to provide comfortable, well-fitting protective eyewear. Modifiable risk factors, such as educational gaps and limited knowledge of occupational hazards, can be addressed to reduce preventable eye injuries among construction workers.

Strengths and limitations

This study provides insight into awareness of occupational ocular injury, utilization of protective eyewear, and factors affecting its use among construction workers. The research group has limited studies in the literature. In addition to assessing the prevalence of awareness and protective practices, this study examined determinants of protective eyewear use using multivariable logistic regression. This study included participants from different occupations within the construction sector, which helped in enhancing the representativeness of the findings. The study identified a few modifiable factors, such as educational status, occupational hazard exposure, type of work, and history of prior eye injury. This could inform the development of future workplace eye safety interventions.

The cross-sectional design was one of the main limitations, as it is not possible to establish causal relationships between protective eyewear use and associated factors with such a study design. The data on ocular injuries, awareness, and protective practices were self-reported and thus may be subject to recall bias. The calculated sample size was not fully achieved; primarily, the study was hospital-based, and there was limited availability of eligible construction workers attending the tertiary care center during the study period. Although these factors may have reduced the statistical power of some subgroup analyses, the sample size of 133 participants was sufficient to conduct statistical analyses, including multivariable logistic regression, and to identify statistically significant predictors of protective eyewear use. Future studies with larger, community-based samples are recommended to validate these findings. One more limitation of a hospital-based study was that the sample may not represent the broader construction worker population, which limits external validity. This poses a potential selection bias, as workers visiting an ophthalmology outpatient department may have better knowledge of ocular hazards or greater prior ocular morbidity relative to the general construction worker population, potentially leading to an overestimation of awareness rates and prior eye injury prevalence. Furthermore, the near-universal level of awareness (99.2%) created a ceiling effect, which precluded meaningful statistical evaluation of the association between awareness and protective eyewear use due to insufficient variability in this variable. As the study was conducted in a specific geographic area and occupational group, the results may limit generalizability. Also, a few subgroup analyses had small sample sizes, which led to wide confidence intervals and decreased estimate accuracy. Although the questionnaire was developed from prior studies in the literature and was expert-reviewed and content-validated by an institutional ophthalmology team, the lack of a formal pilot study has been acknowledged as a limitation. This study did not utilize a formal KAP instrument or composite scoring system; thus, the findings should be regarded as a descriptive characterization of specific awareness and practice items rather than a whole KAP evaluation. The absence of formal dummy-variable coding for work type is also a significant methodological limitation. Work categories in this study are inherently nominal; treating them as ordinal in the regression model assumes equal incremental differences in hazard exposure between categories, which is not statistically justified. This limits the interpretability of the type-of-work coefficient and may have introduced bias into the regression estimate for this variable. Future studies should use dummy variable coding for work type to obtain valid category-specific odds ratios. A further limitation is the handling of multiple-response questions. For questions that permitted more than one response, participants' selected multiple options were collapsed into a single combined category for descriptive display. This approach resulted in the loss of information regarding which specific response combinations were most prevalent and limits the interpretation of these responses.

## Conclusions

This study concluded that although workers' awareness of the use of protective eye gear was high, adherence and use were very low. Educational status, type of work, and prior history of ocular injury were key determinants of protective eyewear use. A few limiting factors, such as poor visibility and discomfort, were associated with non-adherence to protective eyewear use. These findings suggested that there is a need for targeted educational programs, the availability of proper protective equipment, and strict workplace safety policies to increase adherence to protective gear and reduce occupational injuries.

## Appendices

**Table 9:** Questionnaire for assessing awareness, protective eyewear utilization, and associated factors among construction workers. *Multiple answers for these questions are allowed.

Q. no.	Question (English/Hindi)	Responses (English/Hindi)
Awareness domain		
1.	Can your work lead to injury to the eye/क्या आपके काम से आँख को चोट लग सकती	a. Yes/हां, b. No/नहीं, c. Not sure/नहीं पता
2.	Can such an injury lead to blindness/क्या इस तरह की चोट से अंधापन हो सकता है?	a. Yes/हां, b. No/नहीं, c. Not sure/नहीं पता
3.	Ocular injury at your workplace can be prevented by which of following/अपने कार्य स्थल पर आँखों की चोट को निम्नलिखित में से किसके द्वारा रोका जा सकता है*	a. Precaution/एहतियात, b. Eye protection device/आँखों की सुरक्षा वाले चश्मे, c. Keep distance/दूर से काम करके, d. With experience/अनुभव से, e. Other/अन्य
4.	What are various eye protection devices/आँख की सुरक्षा के विभिन्न उपकरण कौनसे है*	a. Normal goggle/glass/spectacle/साधे चश्मा, b. Specialized eye protection gear/glasses/आँखों की सुरक्षा वाले चश्मे, c. Face shield/चेहरे के लिए कवच, d. Others/अन्य
5.	What should be done in case of ocular injury/आँख की चोट के मामले में क्या किया जाना चाहिए*	a. Eye wash/आँख धोना, b. Rubbing of eye/आँख को रगड़ना, c. Over the counter medication/दूकान से ली हुई दवाई
6.	Which doctor, according to you, should be consulted after injury/चोट के बाद आपको किस डॉक्टर से सलाह लेनी चाहिए*	a. Eye specialist/नेत्र विशेषज्ञ, b. General physician/जनरल फिजिशियन, c. Ayurveda/homeopathic practitioners/आयुर्वेद/होमियोपैथिक चिकित्सक, d. Not needed/जरूरत नहीं
Work profile domain		
7.	Type of work/ काम का प्रकार*	a. Hammer-chisel/ छन्नी-हथौड़ा, b. Welding/वेल्डिंग, c. Painting/पुताई, d. Drilling/ड्रिलिंग, e. Stone crushing/पत्थर काटने का काम, f. Others specify/कुछ और
8.	Duration of work/काम की अवधि	a. Less than one year/एक वर्ष से कम, b. 1-5 year/1-5 वर्ष, c. More than 5 years/5 वर्ष से अधिक
9.	Past h/o Fb injury/पहले कभी आंख में चोट लगी थीं क्या	a. Yes/हां, b. No/नहीं
10.	If yes, how many time/यदि हाँ, तो कितने बार	a. 1/1, b. 2/2, c. 3/3, d. More than 3/3 से अधिक
11.	H/o self-medication for the in past injury/पिछले चोट के लिए खुद से दवाई करी थी क्या	a. Yes/हां, b. No/नहीं
12.	Time duration between injury and doctors' visit/चोट और डॉक्टरों के इलाज के बीच का समय	a. <24 h/<24 घंटे, b. 24-72 h/24-72 घंटे, c. >72 h/>72 घंटे
13.	Treating doctor/इलाज करने वाला डॉक्टर	a. Ophthalmologist/नेत्र रोग विशेषज्ञ, b. Non-ophthalmologist/गैर नेत्र रोग विशेषज्ञ
14.	History of injury to other body part/शरीर के अन्य अंग की चोट लगी थी की क्या?	a. Yes/हां, b. No/नहीं
	If yes specify body part affected/यदि हाँ शरीर के प्रभावित हिस्से को निर्दिष्ट करें	
15.	Which treatment was received for the injury/चोट के लिए कहा इलाज करवाए थे	a. Self-medication/खुद से इलाज, b. Hospital treatment/अस्पताल से उपचार
Eye protection practices domain		
16.	Provided with eye protective devices at the workplace?/कार्यस्थल पर नेत्र सुरक्षा उपकरणों को दिया गया है क्या?	a. Yes/हां, b. No/नहीं
17.	Are you using it?/इसका उपयोग कर रहे?	a. Yes/हां, b. No/नहीं
18.	Reason for not using/उपयोग न करने का कारण*	a. No perceived need/कोई ज़रूरत नहीं, b. Poor fitting/खराब फिटिंग, c. Low risk task/कम जोखिम वाला कार्य, d. Short duration of task/कार्य की अल्प अवधि, e. No availability/कोई उपलब्धता नहीं, f. Difficulty in working/ काम करने में कठिनाई, g. Poor visibility/देखने में दिक्कत, h. Forgetfulness/उपयोग करने का याद न रहना, i. Hurry/ जल्दबाज़ी, j. Cost/ कीमत, k. Other/अन्य
